# Patients with Urinary Incontinence Appear More Likely to Develop Upper Urinary Tract Stones: A Nationwide, Population-Based Study with 8-Year Follow-Up

**DOI:** 10.1371/journal.pone.0161223

**Published:** 2016-08-18

**Authors:** Hsiao-Jen Chung, Alex Tong-Long Lin, Chih-Chieh Lin, Tzeng-Ji Chen, Kuang-Kuo Chen

**Affiliations:** 1 Department of Urology, Taipei Veterans General Hospital, Taipei, Taiwan, R.O.C.; 2 Department of Family Medicine, Taipei Veterans General Hospital, Taipei, Taiwan, R.O.C.; 3 Division of Family Medicine, Department of Medicine, National Yang-Ming University, Taipei, Taiwan, R.O.C.; 4 Department of Urology, School of Medicine, Shu-Tien Urological Research Center, National Yang-Ming University, Taipei, Taiwan, R.O.C.; Chinese Academy of Medical Sciences, CHINA

## Abstract

This study aimed to investigate associations between primary urinary incontinence and development of upper urinary tract stones in a nationwide population in Taiwan. Data of 1,777 adults with primary urinary incontinence and 26,655 controls (groups A, B, and C) without urinary incontinence at study inception were retrieved from the National Health Insurance System database in Taiwan and were analyzed retrospectively. No enrolled subjects had previous diagnosis of upper urinary tract stones or spinal cord injury. All subjects were followed through end of 2009, with a minimum follow-up of 8 years. A greater percentage of study subjects (334/1777, 18.8%) developed upper urinary tract stones than that of control groups A (865/8885, 9.7%) and B (888/8885, 10%), and C (930/8885, 10.5%) (all p-values < 0.0001). Urinary incontinence was associated with significantly increased risk of developing urinary tract stones (HR 1.99, 95% CI, 1.70–2.34, p < 0.001). Age and metabolic syndrome status were both associated with developing upper urinary tract stones (both p-values < 0.0001). After adjusting for metabolic syndrome, regression analysis showed that urinary incontinence was still associated with a significantly increased risk of developing upper urinary tract stones (HR 1.99, 95% CI = 1.76–2.26, p < 0.0001). Long-term follow-up of Taiwanese patients with primary urinary incontinence suggests that urinary incontinence is associated with a significantly increased risk of developing upper urinary tract stones. Study findings suggest that physicians treating patients with urinary incontinence should give attention to early detection of upper urinary tract stones.

## Introduction

Primary urinary incontinence (UI) is loss of bladder control, which can be as simple as mild leakage of urine or as severe as uncontrolled voiding. It is commonly associated with aging, and women are affected more than men, primarily pre-menopausal and menopausal women [1]. Primary UI may be caused by weak bladder muscles (stress incontinence), overactive bladder muscles (overactive bladder or urge incontinence), or blockage of an enlarged prostate gland in men. Overweight status, obesity and the presence of metabolic syndrome (MetS) are also associated with primary UI [2,3]. MetS is closely linked to the severity of lower urinary tract symptoms and voiding dysfunction, especially overactive bladder and UI [3,4]. The aggravated renal tubular cell inflammation characteristic of MetS has been suggested as a possible mechanism responsible for stone formation [5]. In community-dwelling Taiwanese men and women, overactive bladder or urge incontinence was reported by 19.8% of subjects and UI was reported by 19.5% of subjects [1]. Some type of UI (stress, urge or mixed) was reported by 69% of Taiwanese women, significantly affecting their quality of life; risk factors were menopause, obesity, and cardiac disease [6]. The prevalence of UI is of great concern, but an even greater concern may be its possible association with development of upper urinary tract stones, as suggested by the dual associations between MetS and UI and between MetS and development of urinary calculi [3].

Nephrolithiasis is associated with high morbidity and high costs, which can create a considerable national healthcare burden [7]. The prevalence of urinary calculi in Taiwan was reported to be 9.6% in 2000, and alcohol consumption and family history of kidney stones were the most significant risk factors [8]. In 2010, an epidemiologic study in a large, nationwide population-based database reported an overall prevalence of urolithiasis of 9.01% [9]. The subtropical temperatures in parts of Taiwan, as well as higher socioeconomic standards due to industrialization and westernization over several decades, have contributed to increased development of urinary tract stones [7]. Previous epidemiologic studies report that water intake, tea drinking and alcohol consumptionmay alter urine composition and thereby increase risk of abnormal crystal formation [10,11]. Climates with higher temperatures also may increase loss of body fluid and reduce urine volume. Excess sun exposure also may lead to endogenous vitamin D production, increased gastrointestinal absorption and urinary excretion of calcium, and eventual formation of calcium oxalate crystals [12]. In addition, high protein, high fat diets are shown to increase risk of urinary stone development [13,14]. However, although multiple causative factors have been identified, the possible association between urinary tract stones and UI has not been studied. The strong evidence-based association between MetS and both of these urinary disorders [3,15] suggest the importance of determining whether a relevant association may exist between UI and upper urinary calculus disease.

The large database of Taiwan’s National Health Insurance System, a nationwide insurance program established in March 1995, gives our research team a perfect opportunity to investigate the association between UI and urolithiasis. Almost the entire population of Taiwan (99%) is covered by this nationwide insurance system, which means that it represents the population across the country. The data have been collected prospectively and protected by the National Health Research Institutes (NHRI) of Taiwan, although data are accessible for research purposes with approval of the NHRI. The insurance system database contains the health records of approximately twenty-six million Taiwanese citizens whose data were accumulated from the beginning of the system to December 2008. Therefore, a large percentage of these patients have longitudinal follow-up of up to eight years—ideal for longitudinal study.

The purpose of this study was to investigate the association between primary urinary incontinence and risk of developing upper urinary tract stones in a nationwide population in Taiwan. To the best of our knowledge, this is the first large-scale study of possible associations between primary UI and urolithiasis.

## Patients & Methods

### Study design

This is a retrospective study conducted using a proprietary secondary database of patients enrolled in the National Health Insurance System, Taiwan. All data for this study were collected prospectively from patients with primary urinary incontinence who were enrolled in the National Health Insurance System in Taiwan between January 1, 1997 and January 1, 2001. Data of patients and controls were collected from the secondary database and were analyzed retrospectively. All enrolled patients had given signed informed consent to the NHRI for their data to be released for research purposes at a later date. Permission and ethical approval were granted to our research team by the National Health Research Institutes (NHRI) of Taiwan to use the extracted data for this study with enrollees’ names encrypted.

### Data source

The Department of Health in Taiwan launched the National Health Insurance Program on March 1, 1995. By the end of 1996, more than 96% of the Taiwanese population was receiving nearly all forms of healthcare services under the program. From the inception of the program, the Bureau of National Health Insurance has prospectively collected patient data, including registration files and original claims for reimbursement. Medical charts and claims are validated by the Bureau to ensure that diagnosis coding is accurate. For research purposes, the National Health Insurance Research Database is derived from this system and is maintained by the National Health Research Institutes (NHRI). The official longitudinal dataset, Longitudinal Health Insurance Database 2005 (LHID 2005), contained a random sample of one million beneficiaries in the year 2005. This dataset represents all beneficiaries and no statistically significant differences are found between the LHID 2005 and all enrollees according to reports of the National Health Research Institute’s. The diagnosis coding system in the database is the International Classification of Diseases (ICD), 9th revision. After receiving ethical approval from the NHRI, we used the LHID 2005 with personal identification encrypted.

### Study subjects and controls

The present study enrolled a total of 1777 adult subjects who had been diagnosed with primary urinary incontinence between 1997 and 2001, and whose data were included in the National Health Insurance System database in Taiwan. The inclusion criteria were: adult patients aged ≥18 years at the time of their enrollment in the National Health Insurance program between January 1, 1997 and January 1, 2001, and patients who had been diagnosed with urinary incontinence (defined as diagnosis of ICD 788.3x or 625.6 with previous prescription of anticholinergic agents or imipramine at least twice since diagnosis). Exclusion criteria were: presence of spinal cord injury (SCI) (defined as diagnosis of ICD 806.xx or 952.xx or 907.2) plus cauda equina syndrome (defined as diagnosis of ICD 344.6x), presence of SCI plus neurogenic bladder (defined as diagnosis of ICD 596.54) and patients with urinary tract stones (defined as diagnosis of ICD 592.x, 594.x, 274.11 or A352) prior to inclusion in the study. Accordingly, no enrolled subjects had a previous diagnosis of upper urinary tract stones or spinal cord injury. The number of radiologic imaging tests in subjects and controls was matched in order to reduce bias. All subjects were followed to the end of 2009 with a minimum follow-up of 8 years.

A total of 17,770 (five controls in each of two control groups for each subject with urinary incontinence) age-, gender- and UI onset date- matched subjects from the same database as the study subjects, but without diagnosis of urinary incontinence, urinary stones or spinal cord injury at baseline, were enrolled as control group A (n = 8885, metabolic syndrome status not matched), control group B (n = 8885, metabolic syndrome status matched) and control group C (n = 8,885, metabolic syndrome status, and X-ray examination rate matched). Controls were randomized conditionally based on the characteristics of the study population. All control subjects were followed to the end of 2009 with a minimum follow-up of 8 years. The control groups were selected in this manner because metabolic syndrome is a risk factor for both urinary incontinence and stones (control group B), and because urologic care is often required or requested for the management of urinary incontinence and it is possible that additional radiologic imaging is obtaining for proper evaluation and management and stones may be found incidentally which will increase the diagnostic rate of stone (control group C matched for X-ray examination results). This method controls for confounders, and while the same patient included in more than one control group, the database is large enough that potential effects of this will not affect the results.

### Definitions

Urinary incontinence was defined as diagnosis of ICD 788.3x or 625.6 with previous prescription of anticholinergic agents or imipramine at least twice since diagnosis. Upper urinary tract stone was defined as diagnosis of ICD 592.xx at least once. Spinal cord injury was defined as diagnosis of ICD (806.xx or 952.xx or 907.2) and (344.60 or 344.61 or 596.54). Metabolic syndrome components were defined as:

(1) DM (250.x or A181); (2) hypertension (401.x–405.x, 437.2 or A269); (3) hyperlipidemia (HLD) (272.0–272.4); (4) heart disease (41x–42x or A278); (5) ischemic stroke (433.x, 434.x, 436, A292 or A293).

Because hypertension and diabetes mellitus (DM) have been shown to be risk factors for urinary stones, these variables were included in multi-variable analysis. Hypertension was defined as diagnosis of ICD 401.xx at least three times within one year. DM was defined as diagnosis of ICD 250.xx at least three times within one year in outpatient service or once in a hospital.

### Statistical analysis

Patients’ characteristics are represented as n (%) or mean ± standard deviations (SD) by group. Differences between groups were compared using Pearson Chi-square test or t-test. Associations between risk factors and the development of upper urinary tract stones were determined using a Cox frailty proportional hazard regression model. A crude model was used to compare the study population’s characteristics, matching sex, age, and UI onset date, and then the crude model was used again in the adjusted model to further match the study population with metabolic syndrome. Results are represented as hazard ratio (HR) with corresponding 95% confidence interval (95% CI). A Cox frailty proportional hazards regression model was used to calculate the risk of upper urinary tract stones between study cohort and control subjects. A Kaplan-Meier curve was used to represent the disease-free rate of upper UTS in patients with and without UI for patients matched with and without on metabolic syndrome. A log-rank test was applied to generate figure data. All data analyses were performed using SAS 9.2 statistics software (SAS Institute Inc., Cary, NC, USA) and R3.1.0 statistical software for Windows. A p value of < 0.05 was considered as statistical significance.

## Results

### Subjects demographic and clinical characteristics

Table 1 represents the demographic and clinical characteristics of 19,547 subjects and controls whose data were evaluated, including 1,777 subjects in the study group (patients with UI), 8,885 subjects in control group A, B, and C, respectively. Analysis revealed that a greater percentage of subjects with UI developed upper urinary tract stones than did those in all control groups without UI (all p-values < 0.0001), specifically, 334 (18.8%) of the study subjects, 865 (9.7%) of control group A subjects, 888 (10%) of control group B subjects, and 930 (10.5%) of control group C developed upper urinary tract stones (Table 1).

**Table 1:** Characteristics of study subjects and controls with and without UI. (N = 28,432).

Variable	Study subjects with UI (n = 1,777)	Control group A^a^ (n = 8,885)	Control group B^b^ (n = 8,885)	Control group C^c^ (n = 8,885)	p-value 1	p-value 2	p-value 3
Sex					1.000	1.000	1.000
Female	1,428 (80.4%)	7,140 (80.4%)	7,140 (80.3%)	7,140 (80.3%)			
Male	349(19.6%)	1,745 (19.6%)	1,745 (19.6%)	1,745 (19.6%)			
Age, years					0.4116	0.4635	0.4498
Mean±STD	57.93±14.7	57.62±15.03	57.65±14.97	57.64±14.97			
Median (Range)	59.54 (21.07,91.11)	58.96 (17.08, 94.09)	59.04 (16.84, 93.83)	59.07 (17.32,94.34)			
Mean difference (UI—No UI)	-	0.3196	0.2843	0.2932			
Metabolic Syndrome					-	1.000	1.000
Yes	183 (10.3%)	-	915(10.3%)	915(10.3%)			
No	1,594 (89.7%)	-	7,970(89.7%)	7,970(89.7%)			
Upper UTS					<0.0001*^+^	<0.0001*^+^	<0.0001*^+^
Yes	334(18.8%)	865(9.7%)	888(10%)	930 (10.5%)			
No	1,443 (81.2%)	8,020 (90.3%)	7,997 (90%)	7955 (89.5%)			

^a^Control group A: an age, gender, UI onset date-matched cohort without UI diagnosis.

^b^Control group B: an age, gender, UI onset date, metabolic syndrome status-matched cohort without UI diagnosis.

^c^Control group C: an age, gender, UI onset date, metabolic syndrome status, and X-ray examination rate-matched cohort without UI diagnosis.

Discrete data are represented as n (%) of patients with(out) UI for given variables; differences between variables were compared using Pearson Chi-square test. Age by group is represented as mean ± STD, median (Range: min, max) of patients with(out) UI; differences between groups in age were compared using t-test. p-value 1, p-value 2, and p-value 3 were derived as compared with Control group A, Control group B, and Control group C. separately.

*^+^ indicates significant difference between patients with UI and cohort groups. (*:p-value < 0.05, ^+^: < 0.0001)

UI, urinary incontinence. UTS, urinary tract stones.

### Associations between subjects’ characteristics and upper urinary tract stones

Tables 2, 3 and 4 represent the associations between upper urinary tract stones and subjects’ characteristics. Results show that sex (compared with control group A, Table 2) and metabolic syndrome status (compared with control B and C, Tables 3 and 4) were both associated with developing upper urinary tract stones (both p-values < 0.0001). (Tables 2, 3 and 4).

**Table 2:** Characteristics of subjects (study group and control group A^a^) with/without development of upper UTS. (N = 10,662)^a^.

variable	Upper UTS (n = 1,199)	Without upper UTS (n = 9,463)	p-value
Sex			<0.0001*^+^
Female	911(10.6%)	7,657 (89.4%)	
Male	288(13.75%)	1,806 (86.25%)	
Age, years			0.2724
Mean±STD	58.08±13.68	57.62±15.13	
Median (Range)	59.20 (17.08, 91.81)	59.01 (17.29, 94.09)	
Difference of mean (UUTS—No UUTS)	-	0.4661	
UI			<0.0001*^+^
Yes	334(18.8%)	1,443(81.2%)	
No	865(9.7%)	8,020(90.3%)	

^a^Control group A: an age, gender, UI onset date-matched cohort without UI diagnosis.

Discrete data, sex and UI are represented as n (%) of patients with(out) upper UTS for given variables; Differences between variables were compared using Pearson Chi-square test. Age by group is represented as mean ± STD, median (Range: min, max) of patients with(out) upper UTS; differences between groups in age were compared using t-test.

*^+^indicates significant association with upper UTS. (*: p-value < 0.05, ^+:^ < 0.0001)

UI, urinary incontinence. UTS, urinary tract stones.

**Table 3:** Characteristics of subjects (study group and control group B^a^) with/without development of upper UTS. (N = 10,662).

variable	Upper UTS (n = 1,222)	Without upper UTS (n = 9,440)	p-value
Sex			0.0784
Female	959(11.2%)	7,609 (89.8%)	
Male	263(12.6%)	1,831 (87.4%)	
Age, years			0.2437
Mean±STD	58.13±13.62	57.64±15.08	
Median (Range)	59.19 (21.07,89.40)	59.12 (16.84,93.83)	
Difference of mean (UUTS—No UUTS)	-	0.4892	
Metabolic Syndrome			<0.0001*^+^
Yes	74(6.7%)	1,024(93.3%)	
No	1,148(12%)	8,416(88%)	
UI			<0.0001*^+^
Yes	334(18.8%)	1,443(81.2%)	
No	888(10.0%)	7,997(90.0%)	

^a^Control B: an age, gender, UI onset date, metabolic syndrome status-matched cohort without UI diagnosis.

Discrete data are represented as n (%) of patients with(out) upper UTS for given variables; Differences between variables were compared using Pearson Chi-square test. Age by group is represented as mean ± STD, median (Range: min, max) of patients with or without UUTS; Differences between groups in age were compared using t-test.

*^+^ indicates significant association with UUTS. (*: p-value < 0.05, ^+:^ < 0.0001)

UI, urinary incontinence. UTS, urinary tract stones.

**Table 4:** Characteristics of subjects (study group and control group C^a^) with/without development of upper UTS. (N = 10,662).

variable	Upper UTS (n = 1,264)	Without upper UTS (n = 9,398)	p-value
Sex			0.0070*
Female	980 (11.4%)	7588 (88.6%)	
Male	284 (13.6%)	1810 (86.4%)	
Age, years			0.0844
Mean±STD	57.05±13.79	57.78±15.07	
Median (Range)	57.72(19.58,89.40)	59.30(17.32,94.34)	
Difference of mean (UUTS—No UUTS)	-	-0.7215	
Metabolic Syndrome			<0.0001*^+^
Yes	74 (6.7%)	1148 (12.0%)	
No	1024 (93.3%)	8416 (88.0%)	
UI			<0.0001*^+^
Yes	334 (26.4%)	1443 (15.4%)	
No	930 (73.6%)	7955 (84.6%)	

^a^ Control group C: an age, gender, UI onset date, metabolic syndrome status, and X-ray examination rate-matched cohort without UI diagnosis.

Discrete data are represented as n (%) of patients with(out) upper UTS for given variables; Differences between variables were compared using Pearson Chi-square test. Age by group is represented as mean ± STD, median (Range: min, max) of patients with or without UUTS; Differences between groups in age were compared using t-test.

*^+^ indicates significant association with UUTS. (*: p-value < 0.05, ^+:^ < 0.0001)

UI, urinary incontinence. UTS, urinary tract stones.

### Time to develop upper urinary tract stones

**Fig 1:**
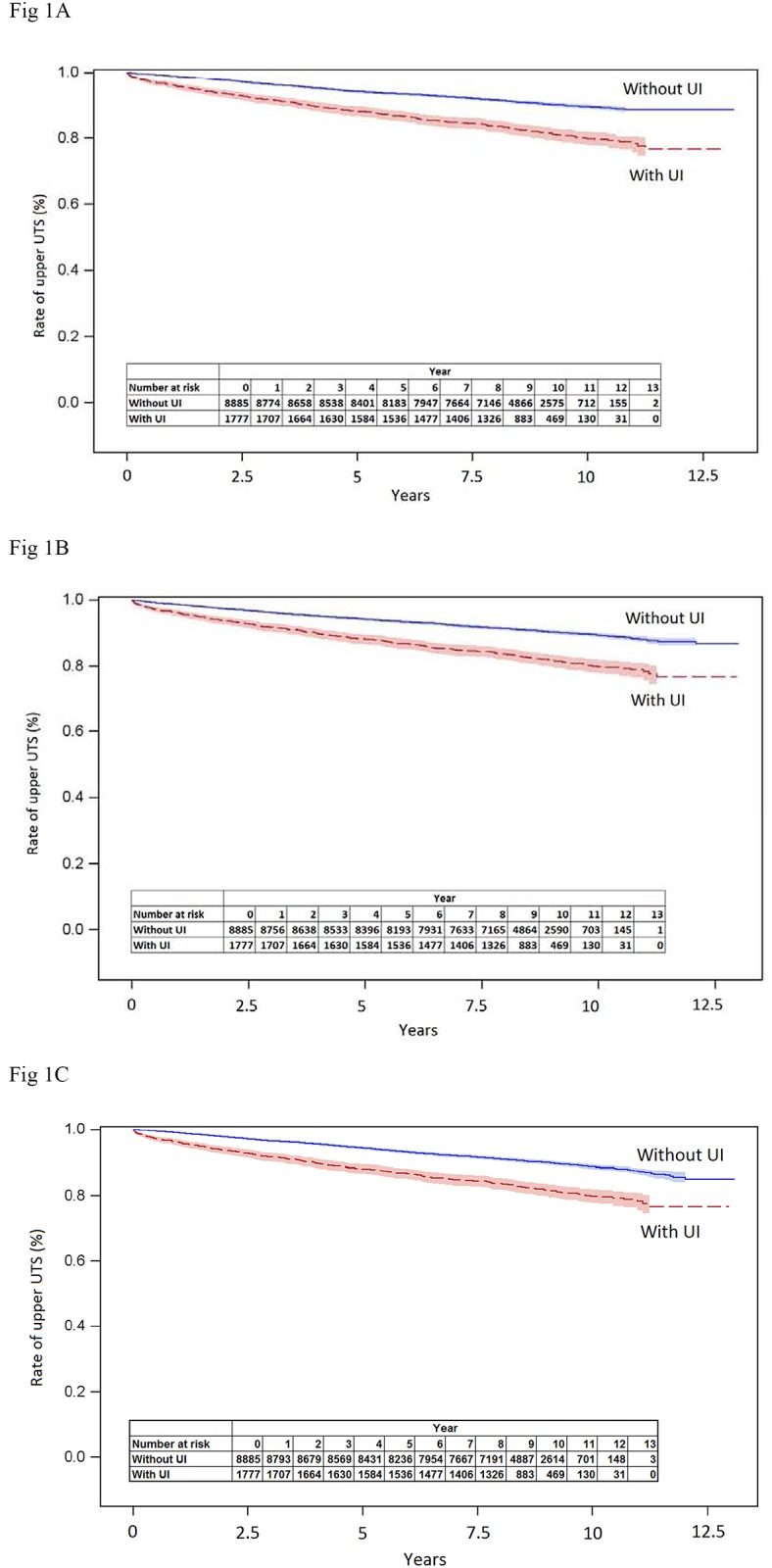
Kaplan-Meier curve of survival rate of developing upper UTS during the follow-up times between patients with UI and without UI for patients were not matched on metabolic syndrome (A) for patients were matched on metabolic syndrome (B), and for patients were matched on metabolic syndrome and X-ray examination (C). In both matching methods, the Log-rank tests show the survival rate of developing upper UTS during the follow-up times were significantly different between patients with and without UI (All p-values < 0.0001).

Regarding the time it takes to develop upper urinary tract stones, subjects’ time without developing upper urinary tract stones (stone-free time) is shown in Fig 1. Table 5 represents the median time to develop upper urinary tract stones, which was observed as: 3.7 years (range: 0.03 months to 11.25 years; 25% [Lower Quartile]:1.26 years, 75% [Upper Quartile]: 6.34 years) for patients with UI and 4.22 (4.15) (4.66) years (range: 0.10 (0.10) (0.03) months to 10.80 (12.09) (12.03) years; 25%: 2.18 (1.85) (2.35) years, 75%: 6.89 (6.86) (7.06) years) for patients without UI, in control groups A(B)(C), respectively.(Table 5, Fig 1A, 1B and 1C).

**Table 5:** Duration free from UTS (years) of subjects with and without UI. (N = 10,662).

	Patients with UI	Control group A^a^	Control group B^b^	Control group C^c^
Variable	(n = 334)	(n = 865)	(n = 888)	(n = 930)
Duration, years				
Median	3.6687	4.2245	4.1506	4.6612
Min	0.0027	0.0082	0.0082	0.0027
25% (Lower Quartile)	1.2567	2.1821	1.8576	2.3491
75% (Upper Quartile)	6.3381	6.8857	6.8569	7.0554
Max	11.2471	10.8008	12.0903	12.0274

^a^Cox frailty proportional hazard regression model was fitted by study group and control group A (matched for age, gender, index ambulatory care visit)

^b^Cox frailty proportional hazard regression model was fitted by study group and control group B (matched for age, gender, index ambulatory care visit, metabolic syndrome)

^c^Control group C: an age, gender, UI onset date, metabolic syndrome status,and X-ray examination rate-matched cohort without UI diagnosis

Log-rank tests showed that the time to develop upper urinary tract stones was significantly different between control group subjects with and without UI (control group A, p < 0.0001; control group B, p < 0.0001; control group C, p<0.0001) by (un)controlling metabolic syndrome status (control groups A, B), and metabolic syndrome status and X-ray examination (control group C), respectively. The Cox frailty proportional hazard regression model fitted based on the study subjects and control group A crude model, also revealed that the development of upper urinary tract stones was associated with UI (HR = 2.06, 95%CI = 1.82–2.34, p<0.0001) (Table 6). After adjusting for the metabolic syndrome, the Cox frailty proportional hazard regression model fitted based on the study subjects and control group B and C, showed that primary urinary incontinence was still associated with a significantly increased risk of developing upper urinary tract stones.

**Table 6:** Association between UI and development of upper UTS (N = 10,662).

	Crude^a^		Adjusted^b^		Adjusted^c^	
	HR (95%CI)	P-Value	HR (95%CI)	P-Value	HR (95%CI)	P-Value
Incontinence	2.06 (1.82–2.34)	<0.0001*^+^	1.99 (1.76–2.26)	<0.0001*^+^	1.94 (1.71–2.19)	<0.0001*^+^

^a^Cox frailty proportional hazard regression model was fitted by study group and control group A (matched for age, gender, index ambulatory care visit)

^b^Cox frailty proportional hazard regression model was fitted by study group and control group B (matched for age, gender, index ambulatory care visit, metabolic syndrome)

^c^Control group C: an age, gender, UI onset date, metabolic syndrome status,and X-ray examination rate-matched cohort without UI diagnosis

HR: hazard ratio; 95%CI: 95% confidence interval of HR.

*^+^indicates significance. (*: p-value < 0.05, ^+:^ < 0.0001)

UI, urinary incontinence. UTS, urinary tract stones.

## Discussion

In the present study, among a total of 1777 adult subjects with UI and 26,655 controls without UI whose data were retrieved from the large database of the National Health Insurance System in Taiwan, a greater percentage of subjects with UI developed upper urinary tract stones than did the controls without UI. Overall, after at least 8 years of follow-up, 334of 1777 study subjects and 2,683 of the 17,770 control subjects in two groups developed upper urinary tract stones. Most importantly, UI was associated with a significantly increased risk of developing urinary tract stones. Among the study subjects, age and MetS status were both associated with developing upper urinary tract stones. The duration of time in the follow-up period for upper urinary tract stones to develop in the control group subjects was significantly different between those who had developed UI and those who did not develop UI, and was dependent on subjects’ MetS status and whether it was controlled or not. After adjusting for MetS, regression analysis showed that primary urinary incontinence was still associated with a significantly increased risk of developing upper urinary tract stones.

Until now, no study has investigated the possible association between urinary incontinence and urinary stones, applying long-term follow-up of a large population-based cohort. The link between the two urinary conditions has been suggested by studies of the association of MetS with urological disease [16], and with increased severity of urological symptoms [3,4]. MetS is not one disease, but a cluster of diseases and interrelated cardiac risk factors, including primarily insulin resistance, hyperglycemia, overweight or obese status and increased BMI, endothelial dysfunction, dyslipidemia and atherosclerosis [17]. It has been linked to urological diseases in multiple studies, including stone formation, an array of lower urinary tract symptoms, urinary incontinence in females, prostatic hyperplasia and prostate cancer, erectile dysfunction and male infertility [15–17]. MetS has not been linked to incontinence in males necessarily [18], although results of our study do support the association between UI and stone development in males. An association has also been demonstrated between obesity and UI, making these both common features of MetS [3,16]. Lee et al. found that central obesity and increased waist circumference were directly associated with and predictive of the severity of voiding dysfunction [3]. In that study, obese males with other MetS components were at increased risk of male pelvic dysfunction. In another study, UI was most prevalent in women with both obesity and diabetes [19]. Gender was not a significant factor in the present study.

The relationship of storage symptoms vs. voiding symptoms is influenced by the sustained hyperglycemia typical of MetS, which acts on neurons in the pelvic ganglion [15]. This may perhaps begin to explain the MetS association with UI, but does not explain stone development. Neither does it explain the association between UI and stone development, although obesity and increased waist circumference are associated with both urinary disorders. Moreover, data from the NHANES III trial showed that increased numbers of MetS components were associated with increased reporting of urinary tract stones [20]. In the present study, among our study subjects with UI, those who had MetS status were most associated with developing upper urinary tract stones; age and MetS were the most significant risk factors. The presence of different MetS components was not specified in the present study, and this remains to be investigated.

Systemic inflammation is a representative characteristic of MetS, with elevated levels of C-reactive protein (CRP), inflamed adipose tissue, and increased secretion of pro-inflammatory cytokines [21]. Zuo et al. [5] found that, in a simulated MetS environment, increased levels of inflammatory cytokines were released, which resulted in increased adhesion of COM crystals to renal tubular epithelial cells and low-grade inflammation via a paracrine mechanism. The authors suggested that such inflammation-mediated injury could then cause a series of events that produce more crystallization and retention, leading to development of urinary tract stones [5]. Although we did not investigate the mechanism behind UI and stone development, it seems plausible that stone development and urinary incontinence could both be influenced by the systemic inflammation common to MetS and specific MetS components.

The prevalence of UI among adults in Taiwan is relatively high, especially among women, and suggests that development of upper urinary tract stones will increase in proportion in this population. A study in Taiwan that used the same nationwide, population-based National Health Insurance Research Database found that there is a high risk (10%) of recurrent urolithiasis in Taiwan [9], but the database only records information of patients actually seeking medical care for stones and stone symptoms. Therefore, the actual numbers of people with or without UI who may have urinary tract stones is relatively unknown. Based on results of the present study, we suggest that one way to identify patients with development and/or recurrence of urinary tract stones is through careful evaluation of all patients with UI and/or MetS. We trust that findings of this study may emphasize careful monitoring of the UI patient base to support early diagnosis and treatment of urolithiasis.

## Limitations

This study has several limitations common to research using a secondary database. First, the results depend entirely on the integrity and accuracy of the database. Although the data were comprehensive in terms of patients’ diagnoses, no information was included about patient diets, lifestyle, living environment, height and weight, and certain laboratory data on metabolic function, which could be important factors related to specific diseases. For this study, we included patients only by diagnostic code (ICD-9) and we cannot know the severity of disease or whether patients were postpartum or not, which could be influential factors. Also, the ICD-9 codes used to identify patients do not distinguish between types of urinary incontinence, and thus the study design did not allow us to determine if any specific type of urinary incontinence is more likely to develop stones. Prospective randomized trials with long-term follow-up are warranted to support the findings of the present study and to further investigate the mechanism responsible for development of upper urinary tract stones in patients with UI.

## Conclusions

Results of the present study suggest that urinary incontinence is an independent risk factor for development of upper urinary tract stones. Although additional studies are warranted, healthcare professionals caring for patients with primary UI may wish to evaluate these patients periodically for early detection and treatment of possible upper urinary tract stones.
